# Rapid Detection of SARS-CoV-2 Using Duplex Reverse Transcription-Multienzyme Isothermal Rapid Amplification in a Point-of-Care Testing

**DOI:** 10.3389/fcimb.2021.678703

**Published:** 2021-10-22

**Authors:** Hui Chen, Chang Sun, Yang Wang, Xiaojiao Gao, Jinwei You, Wanwan Yu, Ning Sun, Yang Yang, Xiaojun Li

**Affiliations:** ^1^ Department of Medicine, JiangSu University, Zhenjiang, China; ^2^ Department of Basic Medical Laboratory, Institute of Clinical Laboratory Science, Jinling Hospital, Clinical School of Medical College, Nanjing University, Nanjing, China; ^3^ Department of Orthopaedics, Jinling Hospital, Clinical School of Medical College, Nanjing University, Nanjing, China; ^4^ Department of Clinical Laboratory, Jinling Hospital, Clinical School of Medical College, Nanjing University, Nanjing, China; ^5^ Department of Laboratory Animal, Jinling Hospital, Clinical School of Medical College, Nanjing University, Nanjing, China; ^6^ Department of Emergency, Jinling Hospital, Clinical School of Medical College, Nanjing University, Nanjing, China; ^7^ State Key Laboratory of Analytical Chemistry for Life Science, Department of Chemistry, Nanjing University, Nanjing, China

**Keywords:** SARS-CoV-2, duplex multienzyme isothermal rapid amplification, point-of-care testing, lateral flow dipstick, nucleic acid tests, without RNA extraction, a low volume

## Abstract

In December 2019, a severe acute respiratory syndrome caused by SARS-CoV-2 spread rapidly worldwide. Portable nucleic acid tests of SARS-CoV-2 are critically important for diagnostics. In this study, we used an isothermal amplification method—Multienzyme Isothermal Rapid Amplification (MIRA)—for rapid detection of SARS-CoV-2. We designed the primers and probes in ORF1ab and N gene of SARS-CoV-2. The amplicons could be monitored by lateral flow dipsticks (LFDs). The reaction temperature, time, concentrations of primers and probes, and working volume were optimized. Four commercial swab collection buffers were used to test the amplification efficacy of our assay without RNA extraction. Our assay was able to amplify duplex targets of SARS-CoV-2 in one single reaction using one-step RT-MIRA. The assay worked well in a low volume of 10 μl at 38°C for 20 min. Using three collection buffers without guanidinium, our assay was able to amplify efficaciously without RNA extraction. The 95% limit of detection (LoD) of the RT-MIRA assay was 49.5 (95% CI, 46.8–52.7) copies/ml for ORF1ab gene and 48.8 (95% CI, 46.5–52.6) copies/ml for N gene. There is no cross-reaction with other human respiratory pathogens, such as SARS-CoV, MERS-CoV, influenza A virus, influenza B virus, human adenovirus, respiratory syncytial virus, human parainfluenza virus, and coronavirus 229E in our assay. The precision evaluation revealed that the C50**−**20% to C50+20% range bounds the C5–C95 interval. This assay also showed high anti-interference ability. The extraction-free RT-MIRA and qPCR detection results of 243 nucleic acid specimens from suspected patients or national references showed a 100.0% (95% confidence interval, 94.2%–100.0%) positive predictive value and a 100.0% (95% confidence interval, 92.7%–100.0%) negative predictive value. Compared with qPCR, the kappa value of the two assays was 1.00 (*P* < 0.0001). In conclusion, we provide a portable and visualized method for detection of SARS-CoV-2 without RNA extraction, allowing its application in SARS-CoV-2 on-site detection.

## Introduction

In early December 2019, Coronavirus disease 2019 (COVID-19), which is caused by beta-coronavirus (SARS-CoV-2), has emerged and became a prevalent viral threat in the world (Wang et al., 2020). By November 17, 2020, there are over 54.4 million cases with over 1,300,000 SARS-CoV-2-related deaths worldwide.

SARS-CoV-2 spread across public community (Rothe et al., 2020; Zhu et al., 2020). Rapid diagnostic testing is needed to prevent infection transmission. Quantitative RT-PCR (qRT-PCR) is a nucleic acid testing method approved for detection of SARS-CoV-2. The current qRT-PCR involves RNA extraction in the pre-analytical phase; thus, the testing time reached 4–6 h and the turnaround time was over 24 h. Detection of SARS-CoV-2 antibody is rapid; however, it takes several days after symptom onset to mount detectable levels of antibody (Zhang et al., 2020). Until now, PCR-based nucleic acid tests are reliable methods for screening SARS-CoV-2 (Wee et al., 2020). The RNA extraction remains a major bottleneck due to the cost and time for the step.

In contrast, isothermal amplifications provided rapid methods for nucleic acid tests (Broughton et al., 2020; Cong et al., 2020). Isothermal amplification such as Helicase-dependent isothermal DNA amplification (HDA), Recombinase Polymerase Amplification (RPA), or Loop-mediated isothermal amplification (LAMP) have been used for detection of viruses including MERS-CoV (Huang et al., 2021), bovine coronavirus (Amer et al., 2013), as well as H7N9 influenza virus (Abd El Wahed et al., 2015) and Ebola virus (Faye et al., 2015). The amplicons could be easily visualized by real-time quantitative PCR (Wang et al., 2018), gel electrophoresis (Hassan et al., 2018), or lateral flow strip assay (Wang et al., 2016). In a previous study, we reported an RT-RPA assay for subtyping of influenza virus (Sun et al., 2018). Isothermal amplification techniques have also been utilized for virus detection in unpurified samples (Nie et al., 2012; Bender et al., 2018; Shen et al., 2019).

Multienzyme Isothermal Rapid Amplification (MIRA) is a new recombinase-based isothermal amplification. Unlike the Recombinase used in RPA (T4 UvsX) or RAA (*E. coli* UvsX) (Zhang et al., 2017), MIRA used the combination of Helicases (gp41) and Recombinase (*Streptomyces coelicolor* recA, SC-recA) to form single-stranded DNA and start the reaction. The multiple enzymes system accelerates the reaction rate and promotes tolerance to potential inhibitors. The amplification process of nucleic acid templates in MIRA could be completed within 10–30 min at 37–42°C. This technique has been used for the detection of swine fever virus (Tu et al., 2021). Similar to other isothermal amplifications, reverse transcription-MIRA (RT-MIRA) products could also be visualized by lateral flow strip assay or real-time quantitative PCR.

Here, we designed duplex one-step RT-MIRA with lateral flow dipsticks (LFDs) for detection of SARS-CoV-2 in ORF1ab gene and N gene without RNA extraction. In our method, heat inactivation, reverse transcription, amplification, and results visualization could be achieved in one tube within 25 min in low volume (10 μl). The limit of detection (LoD) is 49.5 (95% CI, 46.8–52.7) copies/ml for ORF1ab gene and 48.8 (95% CI, 46.5–52.6) copies/ml for N gene. No cross-reaction with other viruses was observed in our assay. By using clinical samples and national references, our assay achieved 100.0% positive predictive value and 100.0% negative predictive value.

## Materials and Methods

### SARS-CoV-2 National Reference

SARS-CoV-2 national reference, which was designed by the National Center for Clinical Laboratories, was used as standard SARS-CoV-2 (Guangdong Hecin Scientific Co., Ltd., China). The national reference contains the full length of RdRp, E, and N gene in SARS-CoV-2 and partial length of ORF1ab and S gene. Target sites of the Chinese Center for Disease Control and Prevention (CDC) and World Health Organization (WHO) were covered. The copy number of national references was quantified using digital droplet PCR. The lyophilized SARS-CoV-2 national references were solubilized in DEPC water following the manufacturer’s instructions before testing. This study was approved by the Ethics Committee of Jinling Hospital (Approval No.: 2020DZWJWKT-005).

### Nucleic Acid Preparation

SARS-CoV and MERS-CoV pseudoviruses were purchased from Fubio Biological Technology Co., Ltd. Influenza A virus (H3N2), influenza A virus (H7N9), and influenza B virus RNA were obtained from Shanghai Institute of Biological Products. Respiratory syncytial virus was isolated by our laboratory. Adenovirus-3, adenovirus-7, parainfluenza virus, and coronavirus 229E were from Health BioMed biomedical Co., Ltd. (Sun et al., 2018). These nucleic acids were for the specificity analysis.

### Primer and Probe Design

According to the manufacturers’ guiding principles, the recommended length of MIRA primers is 30–35 bp. Several pairs of primers specific to the conserved region of ORF1ab gene and N gene of SARS-CoV-2 (RefSeq: NC_045512.2) were manually designed. The specificity of primers was analyzed by using BLAST tools. Reverse primers carried a biotin at the 5’ end for both ORF1ab gene and N gene. The probe for ORF1ab gene included a digoxigenin at the 5’ end. The probe for N gene included a FAM at the 5’ end. Otherwise, the probes contain an internal abasic nucleotide analogue “dspacer” and a C3-spacer at the 3’ end. The sequence of primers and probes was presented in **Supplementary Table S1**. All the oligonucleotides were synthesized by Sangon Biotech Co., Ltd. (Shanghai, China).

### RT-MIRA

RT-MIRA kits were purchased from Weifang Amp-Future Biotech Co. Ltd., Shandong, China. One-step RT-MIRA assays were performed on a 50-μl volume. The mixture was prepared in a tube containing 29.4 μl of buffer A, 2 μl of forward primers (10 μM), 2 μl of backward primers (10 μM), 0.6 μl of probes (10 μM), 10.5 μl of DEPC water, and 3 μl of RNA template. When 2.5 μl of magnesium acetate (280 mM) was added into the 47.5-μl mixture, the reaction was started. The performance of primers was analyzed by 2% agarose gel. The temperature (37, 38, 39, 40, 41, and 42°C) and amplification time (5, 10, 15, 20, 25, 30, and 35 min) were optimized. For duplex amplification, the concentrations of primers and probes were adjusted. For low-volume amplification, the performance of our assay in low volumes was tested by reducing reaction volume to half of the original size (50, 25, 12.5, 6.2, and 3.1 μl). To prepare low reaction volumes, a 50-μl reaction system was placed on ice to prevent MIRA; aliquots of planned low volumes were then transferred into a new individual PCR tubes and then incubated to initiate the reaction.

For the final low-volume reaction system, 29.4 μl of buffer A, primers, and probes rehydrated the lyophilized pellet as previously described in the 50-μl system, and then the amount of amplification reagents that the optimized low volume system acquired was aliquoted into individual PCR tubes. The addition of 3-μl templates and magnesium into the aliquots triggered the low-volume RT-MIRA reaction. DEPC water was used to adjust the reaction system to the final low volume.

For standard RT-MIRA, the gold standard RNA extraction method was prior to the amplification step. According to the manufacturer’s instructions, RNA was extracted from 300 μl of the national references, using the automatic nucleic acid extractor EX3600 (Liferiver Bio-Tech Co., Ltd., Shanghai, China) and the total extracts were tested using the optimized RT-MIRA assay.

For RT-MIRA without RNA extraction, the national references were subjected to 95°C incubation for 2 min, followed by directly loading the inactivated components into the low-volume RT-MIRA reaction system.

### Lateral Flow Dipsticks Readout

LFDs were obtained by a commercial company (Huntarray Biotechnology Co., Ltd., Jiangsu, China). The LFDs contain two detection lines (T line) and one quality control line (C line), fixed with anti-FAM, anti-DIG, and biotinylated mouse anti-rabbit antibodies, respectively. Three microliters of the double-labeled amplicon was diluted into 80 μl of Tris-HCl (50 mM, pH 8.0). After incubating in LFDs for 3 min, LFDs were imaged with a smartphone. The relative intensity of bands was calculated using ImageJ (National Institutes of Health, Bethesda, MD, USA). The threshold value was set according to the sum of 20 PCR negative specimens means and three standard deviations. The specimens would be identified as positive if the relative intensity is over the threshold value (ORF1ab gene, 0.0913; N gene, 0.0782).

### qRT-PCR

The samples were in parallel tested by the standard clinical qRT-PCR assay with a pre-treatment of RNA extraction as described above in the standard RT-MIRA. qRT-PCR kits for SARS-CoV-2 nucleic acid testing were obtained from Liferiver Bio-Tech Co., Ltd., Shanghai, China. qRT-PCR was performed on an Applied Biosystems 7500 real-time PCR system (Thermo Scientific, Waltham, MA, USA). The program was set as follows: pre-amplification 45°C for 10 min, 95°C for 180 s, and 45 cycles of 95°C for 15 s and 58°C for 30 s. The result was identified as positive if cycle threshold (Ct) value < 37.

### Specificity Analysis

Different respiratory viruses, such as SARS-CoV, MERS-CoV, influenza A virus (H3N2), influenza A virus (H7N9), influenza B virus (Flu B), respiratory syncytial virus (RSV), adenovirus-3 (AdV-3), adenovirus-7 (AdV-7), human parainfluenza virus (HPIV), and coronavirus 229E (CoV-229E), were used to evaluate the specificity of our assay without RNA extraction.

### Limit of Detection

The LoD evaluation experiment was performed according to the Clinical and Laboratory Standards Institute (CLSI) EP17-A2 guideline. Seven concentration samples were prepared by diluting low-level SARS-CoV-2 national reference (1.5 × 10^3^ copies/ml) and a set of measurements were made for each concentration (quadruplicate for seven consecutive days). Hit rate at each concentration was calculated: the number of positive/total number of results. Then, all data obtained was reanalyzed for probit analysis and plotted with GraphPad Prism 8.0 (GraphPad Software Inc.). The regression model was applied to determine the concentration corresponding to a 95% hit rate, which would then be identified as the LoD.

### Precision Evaluation

To evaluate the precision, the 1.5 × 10^3^ copies/ml SARS-CoV-2 national references were diluted to a series of low concentrations, which were detected 40 times using our assay to determine the C50 (the concentration generating 35%–65% positive results) according to the CLSI EP12-A2. As the EP12-A2 guidelines emphasized, to verify the precision of a qualitative assay, further research is required to prove whether the C50−20% to C50+20% concentration range bounds the C5–C95 interval (C5, the concentration producing 5% positive results; C95, the concentration producing 95% positive results), and if so, the samples greater than or equal to 20% away from C50 can be expected to yield consistent results.

### Interference Study

The representative endogenous substances, such as whole blood and saliva, and exogenous substances, such drugs as ribavirin and interferon-alpha, were used to confirm the anti-interference performance of our assay. They were separately added into four common collection buffers diluted with or without low titer of lyophilized SARS-CoV-2 national reference.

### Clinical Efficacy and Statistical Analysis

Ninety SARS-CoV-2 PCR-positive nucleic acid specimens were acquired by diluting SARS-CoV-2 national references with DEPC water and divided into high (CT < 20), middle (20 < CT < 30), and low (30 < CT < 37) levels of viral loads (Bruce et al., 2020), pre-identified using the approved qPCR kit from Liferiver Bio-Tech Co., Ltd., and 153 PCR-negative samples from suspected patients collected from January to December 2020 were subjected to the RT-MIRA assay without RNA extraction. To compare our assay to qPCR, positive predictive value, negative predictive value, and kappa value were evaluated by SPSS 22.0 (IBM, Armonk, NY).

## Results

### Design of RT-MIRA

**Figure 1:**
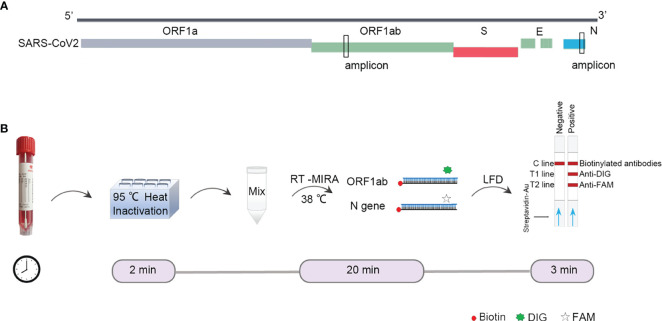
Design of RT-MIRA-based assay for detection of SARS-CoV-2. **(A)** Alignment of the amplicon with SARS-CoV-2. **(B)** Schematic of duplex RT-MIRA and lateral flow dipsticks readout for detection of SARS-CoV-2. C line, Quality Control; T1 line, anti-DIG; T2 line, anti-FAM.

First, we designed the primers targeting ORF1ab gene and N (nucleoprotein) gene of SARS-CoV-2 (**Figure 1A**). The amplified regions overlap target sites selected by WHO. The binding pad was labeled with biotin-conjugated colloidal gold. The T1 line was labeled with anti-DIG, the T2 line was labeled with anti-DIG antibody, and the quality control line was labeled with biotinylated mouse anti-rabbit antibodies. The primers and probes were modified so that the amplicons could be monitored by LFDs. The SARS-CoV-2 test would be positive if both ORF1ab and N gene signals were above the threshold value at the test line 1 and 2, and captured a signal at the control line (**Figure 1B**).

### Screening for Primers

**Figure 2:**
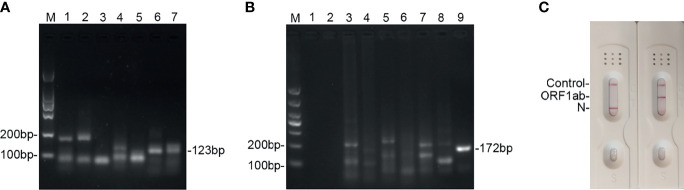
Screening of the primers and probes’ candidates for detection of SARS-CoV-2 by using RT-MIRA. **(A)** Two percent agarose electrophoresis analysis of seven primer pairs used to amplify the ORF1ab gene at 38°C for 30 min. **(B)** Two percent agarose electrophoresis analysis of nine primer pairs used to amplify the N gene of SARS-CoV-2 at 38°C for 30 min. **(C)** LFD readout of amplicons of ORF1ab gene and N gene using probes and primers. SARS-CoV-2 national reference at 5 × 10^4^ copies/ml was used as template.

To screen the optimal primers, the national reference (5 × 10^4^ copies/ml) was used as the template. After RNA extraction, the amplicons were amplified using the basic RT-MIRA and analyzed by 2% agarose gel. The primers with the highest sensitivity and specificity were selected for further analysis. As shown in **Figures 2A, B**, #6 primer for ORF1ab and #9 for N gene had the highest amplification efficacy with expected product sizes. The sequences of primers and probes were presented in **Supplementary Table S1**. When the probes were added into the amplification system, amplicons were able to be monitored by LFDs (**Figure 2C**).

### Optimization of the Standard Duplex RT-MIRA

**Figure 3:**
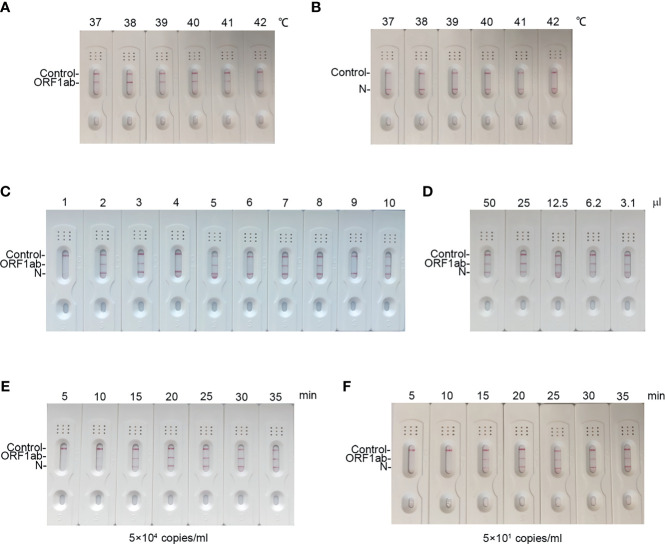
Optimization reaction condition of our assay. **(A, B)** LFD readout of amplicons of ORF1ab gene and N gene in different temperatures ranging from 37°C to 42°C for 30 min using RT-MIRA. **(C)** LFD readout of amplicons of ORF1ab gene and N gene in different concentrations of primers and probes at 38°C for 30 min using RT-MIRA. **(D)** LFD readout of amplicons of ORF1ab gene and N gene in twofold diluted volumes of RT-MIRA at 38°C for 30 min. SARS-CoV-2 national reference at 5 × 10^4^ copies/ml were used as template in above experiments. **(E, F)** LFD readout of RT-MIRA amplicons in different reaction times at 5 × 10^4^ copies or 5 × 10^1^ copies/ml.

To explore the reaction temperature, RT-MIRA was performed at different reaction temperatures ranging from 37°C to 42°C. Compared to other reaction temperatures, reaction temperature at 38°C gained the highest intensity signal (**Figures 3A, B**). The temperature of RT-MIRA was set at 38°C.

Next, we optimized the duplex assay by using different concentrations of primers and probes (**Supplementary Table S2**). As shown in **Figure 3C**, when the concentration of primers was at 200 nM and the concentration of probes was at 100 nM, the amplification system for ORF1ab and N gene gained equal amplification efficacy.

To evaluate the effect of the volume on amplification efficacy, we performed RT-MIRA in twofold diluted volumes. The results demonstrated that a low volume (3.1 μl) has no effect on the efficacy of RT-MIRA (**Figure 3D**). Low volume (3.1μl) of RT-MIRA enables our assay to be used in a microfluid system.

Furthermore, we explored the shortest amplification time of RT-MIRA. RNA with 5 × 10^4^ or 5 × 10^1^ copies/ml was amplified in different amplification times ranging from 5 to 35 min. The intensity of signaling at high levels (5 × 10^4^ copies/ml) was beyond the threshold value, when time is up to 10 min. The intensity of signaling at low levels (5 × 10^1^ copies/ml) was beyond the threshold value, when time is up to 15 min (**Figures 3E, F**).

### Efficient Detection of Our Assay Without RNA Extraction

**Figure 4:**
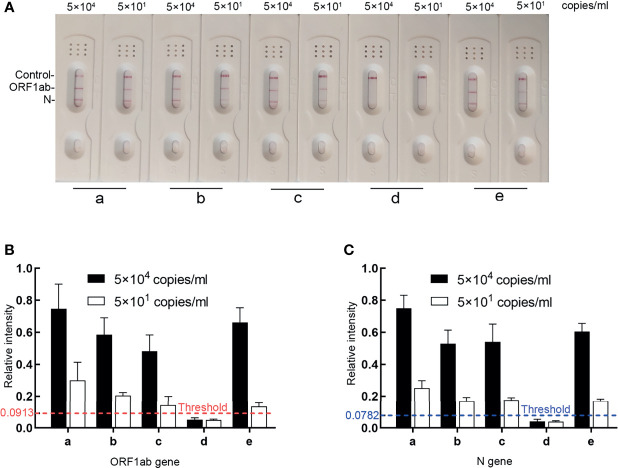
The effects of four collection buffers on amplification efficacy. **(A, B)** LFD readout analysis of RT-MIRA amplicons in different collection buffers. SARS-CoV-2 RNA with 5 × 10^4^ copies or 5 × 10^1^ copies/ml were used as template. **(C)** Relative intensity signaling of the bands was calculated by ImageJ software. a, the extracted SARS-CoV-2 RNA in DEPC water; b, SARS-CoV-2 national reference in UTM1; c, SARS-CoV-2 national reference in UTM2; d, SARS-CoV-2 national reference in media with 30% guanidine; e, SARS-CoV-2 national reference in UTM3.

To further shorten the turnaround time, the RT-MIRA assay was optimized to avoid the RNA extraction step substituted by heat inactivation and reduce the reaction volume. To analyze the effect of collection buffers on amplification efficiency, four swab collections were loaded with high levels (5 × 10^4^ copies/ml) or low levels (5 × 10^1^ copies/ml) of national reference. These diluted samples were pretreated with incubation at 95°C for 2 min and then tested by the optimized extraction-free assay. The extracted nucleic acid of national reference was used as the positive control. Three collections without guanidinium have no effect on amplification efficiency. Collection buffers without guanidinium can be applied in RT-MIRA without RNA extraction (**Figures 4A–C**).

### LoD and Specificity of the Extraction-Free RT-MIRA

**Figure 5:**
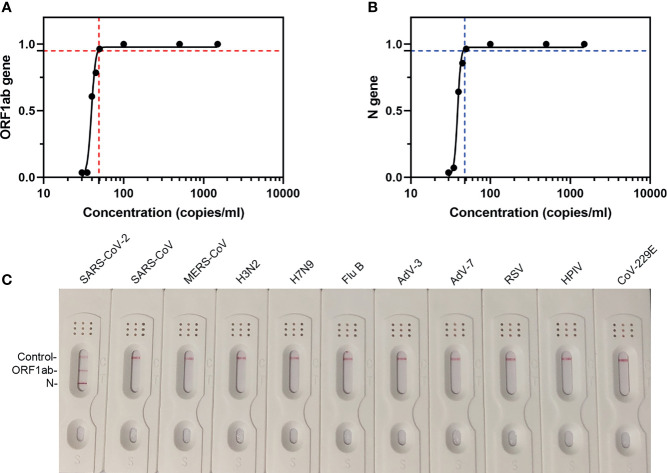
The LoD and specificity of our assay. **(A)** LFD readout analysis of RT-MIRA amplicons in 10-fold diluted SARS-CoV-2 national references. **(B)** Probit analysis for the 95% LoD. The plot illustrated the relationship between the measurand concentration and probability of detection. Seven measurand concentration samples were prepared by diluting low-level SARS-CoV-2 national reference (1.5 × 10^3^ copies/ml). The analyte concentrations were as follows: 0, 30, 35, 40, 45, 50, 100, 500, and 1500 copies/ml. Red-dashed line indicates the 95% LOD of ORF1ab gene. Blue-dashed line indicates the 95% LOD of N gene. **(C)** LFD readout analysis of RT-MIRA amplicons for detection of SARS-CoV-2. H3N2, influenza A virus H3N2; H7N9, influenza A virus H7N9; Flu B, influenza B virus; RSV, respiratory syncytial virus; AdV-3, adenovirus-3; AdV-7, adenovirus-7; HPIV, human parainfluenza virus; CoV-229E, coronavirus 229E.

The LoD of our extraction-free assay was predicted by testing the samples spiked with SARS-CoV-2 national reference at concentrations of 30–1,500 copies/ml. The results are summarized in **Table 1** and the graph of probit analysis is shown in **Figure 5**. According to the fitted probit model, the estimate of 49.5 (95% CI, 46.8–52.7) copies/ml could be taken as the LoD for ORF1ab gene (**Figure 5A**) and the estimate of 48.8 (95% CI, 46.5–52.6) copies/ml could be taken as the LoD for N gene (**Figure 5B**).

**Table 1:** The percentage of positive test results at each diluted concentration.

Concentration (copies/ml)	Positive/Total Results		Hit Rate	
	ORF1ab gene	N gene	ORF1ab gene	N gene
0	0/28	0/28	0.000	0.000
30	1/28	1/28	0.0357	0.0357
35	1/28	2/28	0.0357	0.0714
40	17/28	18/28	0.607	0.643
45	22/28	24/28	0.785	0.857
50	27/28	27/28	0.964	0.964
100	28/28	28/28	1.000	1.000
500	28/28	28/28	1.000	1.000
1,500	28/28	28/28	1.000	1.000

To determine the specificity of this assay, RT-MIRA has been developed to detect eight other respiratory viruses’ nucleic acids. Results showed that this assay did not cross-react with other respiratory viruses (**Figure 5C**).

### Precision Evaluation

The prepared C50 was 40 copies/ml in our assay, at which 22 out of 40 (55%) positive results were observed. This value met EP12-A2 guidelines. The analyte concentrations, including C50−20%, C50, and C50+20%, was repeatedly tested 40 times. The precision results of our assay without RNA extraction are presented in **Table 2**. The +20% samples produced 38/40 to 39/40 (95.0%–97.5%) positive results and the −20% samples produced 39/40 to 40/40 (97.5%–100.0%) negative results. The C50−20% to C50+20% concentration range encompasses C5–C95 interval.

**Table 2:** The precision results of our RT-MIRA assay without RNA extraction.

Gene	Positive/total results at C50	Positive/total results at C50+20%	Negative/total results at C50−20%
ORF1ab gene	22/40 (0.550)	38/40 (0.950)	40/40 (1.000)
N gene	22/40 (0.550)	39/40 (0.975)	39/40 (0.975)

### The Anti-Interference Ability

**Figure 6:**
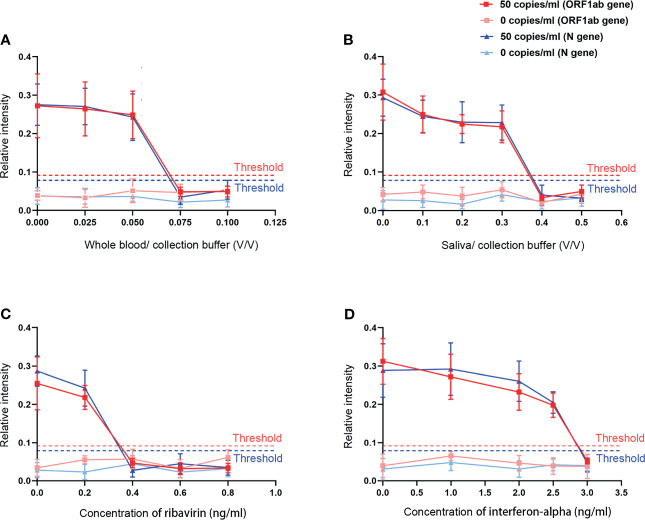
The effects of different interfering substances on our assay. **(A)** Whole blood. **(B)** Saliva. **(C)** Ribavirin. **(D)** Interferon-alpha.

To estimate the selectivity of this extraction-free assay for SARS-CoV-2 detection, we added different concentrations of the most possible coexistence substances in swab samples including the whole blood and saliva and drugs like ribavirin and interferon-alpha into the guanidinium-free collection buffer for the interference study. To avoid false-positive and false-negative results, we compared the test results of the buffer loaded with and without SARS-CoV-2 as 50 copies/ml. The results indicated that the addition of whole blood (<5%) and saliva (<30%) did not influence the direct determination of SARS-CoV-2. At reasonable drug concentrations (ribavirin < 0.2 ng/ml; interferon-alpha < 2.5 ng/ml), there was no interference effects obtained on the detection of SARS-CoV-2 (**Figure 6**).

### Clinical Sample Evaluation

We assessed the performance of the direct RT-MIRA assay for detection of SARS-CoV-2 nucleic acid in clinical samples. A total of 153 PCR-negative specimens in crude matrices were from patients with acute respiratory syndrome. The lyophilized national reference was solubilized in different volumes of DEPC water to acquire positive specimens. We divided these positive specimens into three groups (each 30), based on their final CT values identified by the approved qPCR kit, to test the performance of our assay. None of the 153 PCR-negative specimens were amplified (153/153), providing a negative predictive value of 100% (95% CI, 94.2%–100.0%) of the RT-MIRA assay. The positive predictive value of our assay was 100.0% (95% CI, 92.7%–100.0%). Compared with qRT-PCR, our assay showed a kappa value of 1.00 (*p* < 0.0001) (**Table 3**).

**Table 3:** The performance of RT-MIRA for detection of SARS-CoV-2 in clinical samples or national references.

Samples		RT-MIRA without RNA extraction		RT-MIRA with RNA extraction		qPCR	
		+	-	+	-	+	-
High (*n* = 30)	+	30	0	30	0	30	0
	–	0	0	0	0	0	0
Middle (*n* = 30)	+	30	0	30	0	30	0
	–	0	0	0	0	0	0
Low (*n* = 30)	+	30	0	30	0	30	0
	–	0	0	0	0	0	0
Suspected (*n* = 153)	+	0	0	0	0	0	0
	–	0	153	0	153	0	153
Total (*n* = 243)	+	90	0	90	0	90	0
	–	0	153	0	153	0	153

## Discussion

SARS-CoV-2 is a major pandemic virus that threatens the health of people. The cases of asymptomatic infection increased the pool of individuals who need to be screened. A portable diagnostic method is urgently needed. Due to the need of expensive laboratory instrumentation, qRT-PCR-based assay was restricted to accredited laboratories. Long turnaround times for the whole procedure of RNA extraction and amplification led us to develop a more rapid and reliable method.

Nowadays, multiple isothermal amplification-based methods for detection of SARS-CoV-2 have been developed. Broughton et al. reported a loop-based amplification (RT-LAMP) and CRISPR-Cas12 detection (Broughton et al., 2020). Behrmann et al. reported a real-time RT-RPA assay that could detect SARS-CoV-2 nucleic acid in a low volume (Behrmann et al., 2020). Meanwhile, some researchers (Burton et al., 2021) have devoted their effort to detect SARS-CoV-2 directly on clinical swabs without RNA extraction recently (Eckel et al., 2020; Smyrlaki et al., 2020; Lalli et al., 2021; Taki et al., 2021; Wei et al., 2021). The RT-MIRA has a potential to directly amplify the target genes in crude matrices, which eliminates the RNA extraction step to greatly reduce the assay run time.

In this study, we integrated duplex RT-MIRA with LFDs for detection of SARS-CoV-2. 95°C heating treatment was used to inactivate viruses, release viral RNA, and cause substances that may inhibit the amplification process denatured in the swabs (Smyrlaki et al., 2020). Meanwhile, it has been proved that no viable virus was detected after heating to 95°C for 1 min (Burton et al., 2021). In the study of Batéjat et al., 95°C for 3 min was sufficient to inactivate the virus in clinical samples (Batejat et al., 2021). Thus, 95°C heating could completely inactivate viruses in a short time. We expected that heat inactivation could replace the RNA extraction and the direct RT-MIRA would provide a comparable detection of SARS-CoV-2 to conventional qPCR.

First, our assay has shortened the turnaround time to 20–25 min and simplified the instrument, which is required for nucleic acid amplification. Second, we detected duplex targets (ORF1ab and N gene) of SARS-CoV-2 in one reaction, which improved the specificity of detection. Third, our method was able to work in a low volume (3.1 μl), which means that it might work well in a microfluid system. Considering the feasibility and operability for practical use, thus, 10 μl was chosen as the final reaction volume to develop our assay without RNA extraction.

Fourth, the most common solution to inactivate virus is to denature the viral protein in the chemical denaturants such as guanidinium-base buffer (Burton et al., 2017), but the components could cause potential false-negative cases (Pan et al., 2020a). In our assay, most UTMs without guanidinium were suitable for the amplification without nucleic acid extraction. Moreover, the LoD of our method is comparable to qRT-PCR. The LoD of Chinese CDC assay is at least 500 copies/ml. The analytic performance of the COVID19 ID NOW EUA assay is 125 copies/ml (Mitchell and George, 2020). As shown in **Figures 5A**, **B**, the LoD of our assay is 49.5 copies/ml with no cross-reaction to other viruses.

Considering the bleeding inevitably occurring in the sampling process and the saliva or residual drugs (Song et al., 2017), we investigated the effects of various concentrations of these substances on this SARS-CoV-2 detection system. Our assay showed a good performance when approximately 5% whole blood or 30% saliva were added and could tolerate ribavirin up to 0.2 ng/ml and interferon-alpha up to 2.5 ng/ml. Finally, we compared the efficacy of RT-MIRA and qPCR by using a validation study. The results showed that the two methods had good consistency with a kappa value of 1.00 (*p* < 0.0001). Our assay was proved to be accurate and reliable with a high positive predictive value (100.0%) and negative predictive value (100.0%), respectively. This extraction-free method is, therefore, with high sensitivity, satisfactory specificity, good precision, and anti-interference capacity for SARS-CoV-2 detection.

In comparison to the RPA assay developed by Lau et al. (2021) who report a LoD at 95% probability of 7 copies/μl *in vitro* transcript RNA with a LFD strip, our assay employed the same visualization method, which is of a much lower detection limit, and skipped the RNA extraction step to directly detect the inactivated clinical samples. When compared to the point-of-care testing of SARS-CoV-2 published by Li et al. (2021), who report a detection limit of 25 copies/ml by fabricating an electrothermal heater integrated paper‐based device combined with the colored LAMP technology to visually detect nucleic acid, our assay is of a much simpler design and is a faster approach to obtain results while showing similar analytical sensitivity. There are many sample-to-answer molecular diagnostic platforms under emergency use authorization by FDA such as Cepheid Xpert Xpress SARS-CoV-2, Abbott ID NOW COVID-19, and GenMark ePlex SARS-CoV-2 Test. Zhen et al. (2020) found that ID NOW took the lowest time to yield result (∼17 min) among the three, which is also lower than our assay, but it lost in clinical performance with an unsatisfactory detection limit of 20,000 copies/ml.

As a nucleic acid test, our assay has similar limitations. First, viral titers in patients have no correlation with disease severity (Pan et al., 2020b; Zou et al., 2020). Second, a negative nucleic acid test does not exclude SARS-CoV-2 infection. Only about 70% of patients with SARS-CoV-2 infection are positive in nucleic acid test (Kucirka et al., 2020). Combination nucleic acid test with serologic test or CT (computed tomography) test is needed for clinical suspicion patients.

In conclusion, we report a duplex RT-MIRA-LFDs assay that can be used to detect SARS-CoV-2 nucleic acid in a single tube. Due to the fixed reaction temperature (38°C) and rapid turnaround time (25 min), this assay could enable point-of-care testing outside the clinical diagnostic laboratory.

## Data Availability Statement

The original contributions presented in the study are included in the article/**Supplementary Material**. Further inquiries can be directed to the corresponding authors.

## Ethics Statement

This study was approved by the Ethics Committee of Jinling Hospital (Approval No. 2020DZWJWKT-005).

## Author Contributions

YY, CS, YW, XG, and HC designed the study and analyzed data. HC performed the experiments. YY, XG, JY, WY, NS, and XL edited and reviewed the manuscript. All authors contributed to the article and approved the submitted version.

## Funding

This work was funded by the Jiangsu Postdoctoral Research Foundation (2018K280C to YY), the China Postdoctoral Science Foundation (2020M670092ZX to YY), the Research Fund of Jiangsu health and Health Committee (M2020029 to YY), the Research Fund of Laboratory Animal of Army [SYDW (2020)-17], and the National Key Clinical Program of China (2014ZDZK003 to XL).

## Conflict of Interest

The authors declare that the research was conducted in the absence of any commercial or financial relationships that could be construed as a potential conflict of interest.

## Publisher’s Note

All claims expressed in this article are solely those of the authors and do not necessarily represent those of their affiliated organizations, or those of the publisher, the editors and the reviewers. Any product that may be evaluated in this article, or claim that may be made by its manufacturer, is not guaranteed or endorsed by the publisher.
