# Supplementary material for: Rapid Detection of SARS-CoV-2 Using Duplex Reverse Transcription-Multienzyme Isothermal Rapid Amplification in a Point-of-Care Testing
Source: Front Cell Infect Microbiol. 2021 Oct 22;11:678703. doi: 10.3389/fcimb.2021.678703 (PMC8569318; doi:10.3389/fcimb.2021.678703)
Supplement: Supplementary file 1 [file Table_1.docx]

**Supplemental table 1.** Primers and probes for detection of SARS-CoV-2 in this study

| **Gene** | **Name** | **Sequence（5’-3’）** |  |
| --- | --- | --- | --- |
| N gene | N F | AAGCCTCTTCTCGTTCCTCATCACGTAGTCGC |  |
|  | N R | Biotin-CCTTTACCAGACATTTTGCTCTCAAGCTG |  |
|  | N P | FAM- ATGGCGGTGATGCTGCTCTTGCTTTGCTGC/idSp/GCTTGACAGATTGAAC -C3 Spacer |  |
|  |  |  |  |
|  |  |  |  |
| ORF1ab | ORF1ab F | CTAATGACCCTGTGGGTTTTACACTTAA |  |
|  | ORF1ab R | Biotin-ATTGTGCATCAGCTGACTGAAGCATGGGTTCGC |  |
|  | ORF1ab P | Dig- TCTGTACCGTCTGCGGTATGTGGAAAGGTTA/idSp/GGCTGTAGTTGTGATCA- C3 Spacer |  |
|  |  |  |  |
|  |  |  |  |
