# Supplementary material for: Rapid Detection of SARS-CoV-2 Using Duplex Reverse Transcription-Multienzyme Isothermal Rapid Amplification in a Point-of-Care Testing
Source: Front Cell Infect Microbiol. 2021 Oct 22;11:678703. doi: 10.3389/fcimb.2021.678703 (PMC8569318; doi:10.3389/fcimb.2021.678703)
Supplement: Supplementary file 2 [file Table_2.docx]

**Supplemental table 2.** Different concentrations of primers and probes used in Fig.3C

| **Primer or Probe** | **Concentration (nM)** | | | | | | | | | |
| --- | --- | --- | --- | --- | --- | --- | --- | --- | --- | --- |
|  | **1** | **2** | **3** | **4** | **5** | **6** | **7** | **8** | **9** | **10** |
| ORF-F | 50 | 100 | 200 | 300 | 400 | 50 | 100 | 200 | 300 | 400 |
| ORF-R |  |  |  |  |  |  |  |  |  |  |
| ORF-P | 80 | 80 | 80 | 80 | 80 | 100 | 100 | 100 | 100 | 100 |
| N-F | 200 | 200 | 200 | 200 | 200 | 200 | 200 | 200 | 200 | 200 |
| N-R |  |  |  |  |  |  |  |  |  |  |
| N-P | 80 | 80 | 80 | 80 | 80 | 100 | 100 | 100 | 100 | 100 |
